# The Roles of Drug Metabolism-Related ADH1B in Immune Regulation and Therapeutic Response of Ovarian Cancer

**DOI:** 10.3389/fcell.2022.877254

**Published:** 2022-06-09

**Authors:** Zhijie Xu, Bi Peng, Fanhua Kang, Wenqin Zhang, Muzhang Xiao, Jianbo Li, Qianhui Hong, Yuan Cai, Wei Liu, Yuanliang Yan, Jinwu Peng

**Affiliations:** ^1^ Department of Clinical Laboratory, Xiangya Hospital, Central South University, Changsha, China; ^2^ Department of Pathology, Xiangya Hospital, Central South University, Changsha, China; ^3^ Department of Pathology, Xiangya Changde Hospital, Changde, China; ^4^ National Clinical Research Center for Geriatric Disorders, Xiangya Hospital, Central South University, Changsha, China; ^5^ Department of Burn and Plastic Surgery, Xiangya Hospital, Central South University, Changsha, China; ^6^ Department of Orthopedic Surgery, The Second Hospital University of South China, Hengyang, China; ^7^ Department of Pharmacy, Xiangya Hospital, Central South University, Changsha, China

**Keywords:** ovarian cancer, ADH1B, drug metabolism-related genes, immune infiltration, therapeutic response

## Abstract

**Background:** The different pharmacological effects of drugs in different people can be explained by the polymorphisms of drug metabolism-related genes. Emerging studies have realized the importance of drug metabolism-related genes in the treatment and prognosis of cancers, including ovarian cancer (OV). In this study, using comprehensive bioinformatics and western blot, we identified that the drug metabolism-related gene, ADH1B, was significantly down-regulated in OV cells and tissues. The patients with a high level of ADH1B presented a good prognosis. We also found a negative correlation between ADH1B expression and the activity of chemotherapeutic agents, such as cyclophosphamide. In addition, positive correlations were observed between ADH1B expression and multiple immune checkpoints, including LAG3 and HAVCR2. The immune infiltration analysis further indicated that aberrantly expressed ADH1B might have important roles in regulating the infiltration of macrophages and neutrophils in OV tissues. Then, the co-expression analysis was conducted and the top three enriched KEGG pathways were spliceosome, RNA transport, and DNA replication. In conclusion, the drug metabolism-related gene ADH1B and its interactive network play an essential role in the immune regulation and therapeutic response and maybe identified as promising therapeutic targets for OV patients.

## Introduction

Ovarian cancer (OV) is one of the most frequently diagnosed and the most lethal type of cancer among all gynecological malignancies throughout the world (Siegel et al., 2021). Moreover, high-grade serous ovarian cancer is the most common and invasive histological type of epithelial ovarian cancer (Govindarajan et al., 2020). Despite advances in surgery, chemotherapy, and immunotherapy (Kuroki and Guntupalli, 2020), survival in patients with OV has stagnated for decades, mainly due to diagnosis at an advanced stage and rapidly increasing prevalence of resistance (Muñoz-Galván et al., 2020). Therefore, it is imperative to provide a promising new therapeutic strategy to improve the outcomes of OV patients.

Pharmacogenomics (PGx) is the study of the mechanism between genes and drug metabolism to predict efficacy and relieve side effects (Wake et al., 2019). With the progress of medicine, the idea that genetic variations could be used to personalize drug treatment become possible (Roden et al., 2019). Drug metabolism genes, as the name suggests, are genes that affect drug metabolism. Examples include that mutation in the DPYD gene cause more severe toxicity in patients treated with fluorouracil and loss-of-function alleles in the NUDT15 gene induce a reduction in the degradation of active nucleotide metabolites and result in myelosuppression (Henricks et al., 2018; Relling et al., 2019).

Alcohol dehydrogenase 1B (ADH1B) is located on human chromosome 4q21-q23 and its primordially well-known function is alcohol metabolism (Strommer, 2011; Wei et al., 2021). To date, emerging evidence has demonstrated that ADH1B-associated signaling pathways are key factors for tumorigenesis and progression, including esophageal squamous cell cancer (ESCC) (Suo et al., 2019) and colorectal cancer (CRC) (Choi et al., 2021). Nevertheless, the research on ADH1B and ovarian cancer are much less. It is a discussible question about the roles of ADH1B on prognosis and immune infiltration in ovarian cancer.

To reveal the important role of ADH1B in the treatment of ovarian cancer, our work mainly focuses on exploring the clinical and immunotherapeutic implications of it. First, several public databases were used to demonstrate that aberrantly expressed ADH1B influences the progression and prognosis of ovarian cancer. Furthermore, we identified the expression levels of ADH1B in ovarian cancer from several aspects. Then, the potential signaling pathways and biological functions of ADH1B were found through KEGG and GO annotation. Additionally, the correlations between ADH1B and tumor-infiltrating immune cells (TIICs) and immune checkpoints in ovarian cancer were deeply studied for an underlying immunotherapy target and ready-made targeted drugs. These results provide a novel and significant insight into the treatment of ovarian cancer.

## Methods

### Data Extraction

Datasets of ovarian cancer were obtained from the gene expression omnibus (GEO) database which contained a lot of clinical information and all drug metabolism-related genes. We ultimately identified two ovarian cancer datasets (GSE26712 and GSE18520) (Mok et al., 2009; Vathipadiekal et al., 2015) according to the following conditions: 1) cancer ype: ovarian cancer, 2) study type: expression profiling by array, and 3) analysis type: umor tissue vs. normal tissue. Furthermore, differentially expressed genes (DEGs) were screened out (*p*-value <0.01 and | log FC | >3), which got ready for Venn diagram. Then, we obtained the intersection that is co-differentially expressed genes (co-DGEs) between DEGs and drug metabolism-related genes.

### Prognostic Analysis

The Kaplan-Meier plotter is a web-based tool to evaluate clinical outcomes and explore survival biomarkers. It currently contains more than 25,000 samples from 21 tumor types (Lánczky and Győrffy, 2021). We utilized the Kaplan-Meier plotter to assess the correlation between the expression of co-DEGs and the patients’ prognosis, including overall survival (OS) and progressive survival (PPS).

### Gene Expression Analysis

Multiple databases were used for the evaluation of ADH1B expression levels in ovarian cancer. The Cancer Genome Atlas (TCGA) is a free data collection library which includes the clinicopathology and molecular expression information from 33 types of tumors (Liu et al., 2018). We extracted the expression data of 427 ovarian cancer tissues and 88 normal tissues from it for further validation. The ADH1B expressed levels between tumor group and normal control were analyzed to take advantage of GSE26712 and GSE18520 which were two datasets related to ovarian cancer from the GEO database. Furthermore, these results gave a further verification of ADH1B expression levels using the TCGA database, TNM plot, and GEPIA2 database. TNM plot, a convenient website, contains gene arrays from the GEO and RNA-seq data from TCGA and makes a comparison of the gene expressed levels in normal, tumor, and metastatic groups (Bartha and Győrffy, 2021). And GEPIA2, an updated and enhanced web, be appropriate for retrieving massive gene expression profiles and conducting interaction analysis (Tang et al., 2019).

### Cell Culture

We collected human ovarian epithelial cells IOSE80 and ovarian cancer cells A2780 and TOV112D from the Center for Molecular Medicine, Xiangya Hospital, Central South University and inoculated them onto Dulbecco’s Modified Eagle’s Medium (DMEM, Gibco, United States) with 10% fetal bovine serum (FBS, Gibco, United States) and 1% penicillin/streptomycin (Gibco, United States). These culture mediums were stored at a temperature of 37 with 5% CO2 in a sterile.

### Western Blot

The experiment began with the addition of RIPA buffer supplemented with a protease inhibitor to cell lysates. The BCA protein assay kit was applied to test total protein concentration and ads-polyacrylamide gel electrophoresis was used to load equal amounts of protein in each lane. In addition, we passed on the bands to the PVDF membranes which were sealed for 1 h at room temperature with TBST and added 5% non-fat dry milk. Subsequently, these membranes were incubated at 4°C overnight with primary antibodies called ADH1B monoclonal antibody (Item no: 66939-1-Ig). At last, we tested protein bands using the Immobilon Western Chemiluminescent HRP reagents.

### Correlation Analysis

LinkedOmics is an efficient platform for the access, evaluation, and comparison of cancer multi-omics data (Vasaikar et al., 2018). Correlation analysis was conducted via the LinkFinder module of this database to screen positively and negatively co-expressed genes, the results were shown in the form of volcano plots and heat maps. Subsequently, gene enrichment analysis was performed through the LinkInterpreter module, gene ontology biological process (GO_BP), gene ontology cellular component (GO_CC), gene ontology molecular function (GO_MF), and Kyoto Encyclopedia of Genes and Genomes (KEGG) pathways were realized.

### Immunological Analysis

The evaluation of the ADH1B expression and 24 types of tumor-infiltrating immune cells (TIICs) in ovarian cancer was finished using the single-sample GSEA (ssGSEA) algorithm. Further validations were realized through Tumor IMmune Estimation Resource 2.0 (TIMER) database contributing to TIICs assessment (Li et al., 2020). More convincingly, we employed the TISIDB database (Ru et al., 2019) to verify these immunological results thirdly. Additionally, immunostimulators, immunoinhibitors, chemokines, and receptors targeting ADH1B expression were also explored with the TISIDB platform.

### Treatment Correlation Analysis

CellMiner Cross-Database (CellMinerCDB), a publicly available web, integrates and analyzes molecular and pharmacological data from the National Cancer Institute’s cancer cell line and cross cell line datasets (Luna et al., 2021). The correlation between chemotherapeutic drug activity and ADH1B expression was analyzed by CellMinerCDB. In addition, the relationship between immune checkpoints and ADH1B expression was evaluated with Spearman’s correlation test.

## Results

### The Identification of Differentially Expressed Genes

**FIGURE 1:**
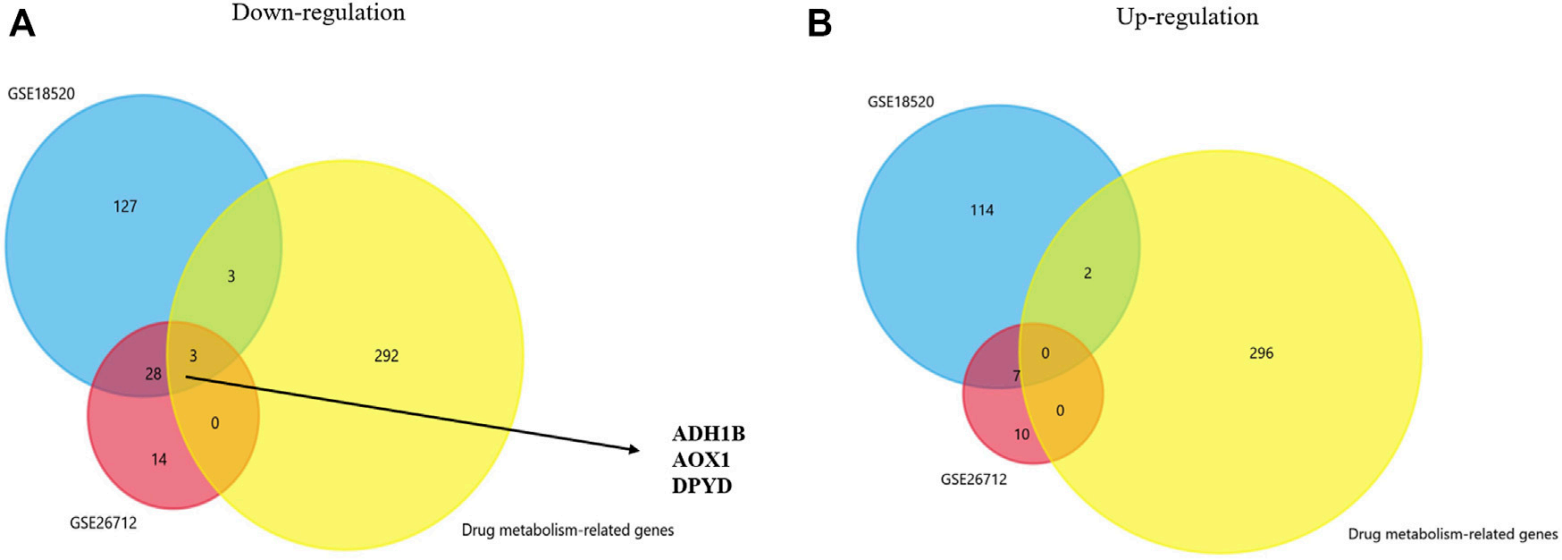
Venn plot for identifying co-DEGs. **(A,B)** Two GEO datasets and the drug metabolism-related gene dataset were used to identify the upregulated and downregulated co-DEGs, respectively.

We analyzed gene expression profiles from GSE18520 and GSE26712 and identified the differentially expressed genes between the normal group and the ovarian cancer group under the filter criteria (*p* <0.01 and | log FC | >3). Then, we found 143 upregulated genes and 161 downregulated genes in GSE18520, and 17 and 35 in GSE26712. In recent years, many studies have suggested that certain genes play a significant role in drug metabolism and disposition and cancer chemical resistance (Mazor et al., 2019; Wang et al., 2020; Akkaif et al., 2021). A genetic approach to reversing chemotherapy resistance could be a new and promising breakthrough. Thus, co-DEGs were obtained from two GEO database and a drug metabolism-related gene dataset by Venn analysis. We initially identified three down-regulated genes whose names were ADH1B, AOX1, and DPYD (Figures 1A,B). Further investigations were focused on their prognostic significance.

### ADH1B Closely Related to the Prognosis of Patients With Ovarian Cancer

**FIGURE 2:**
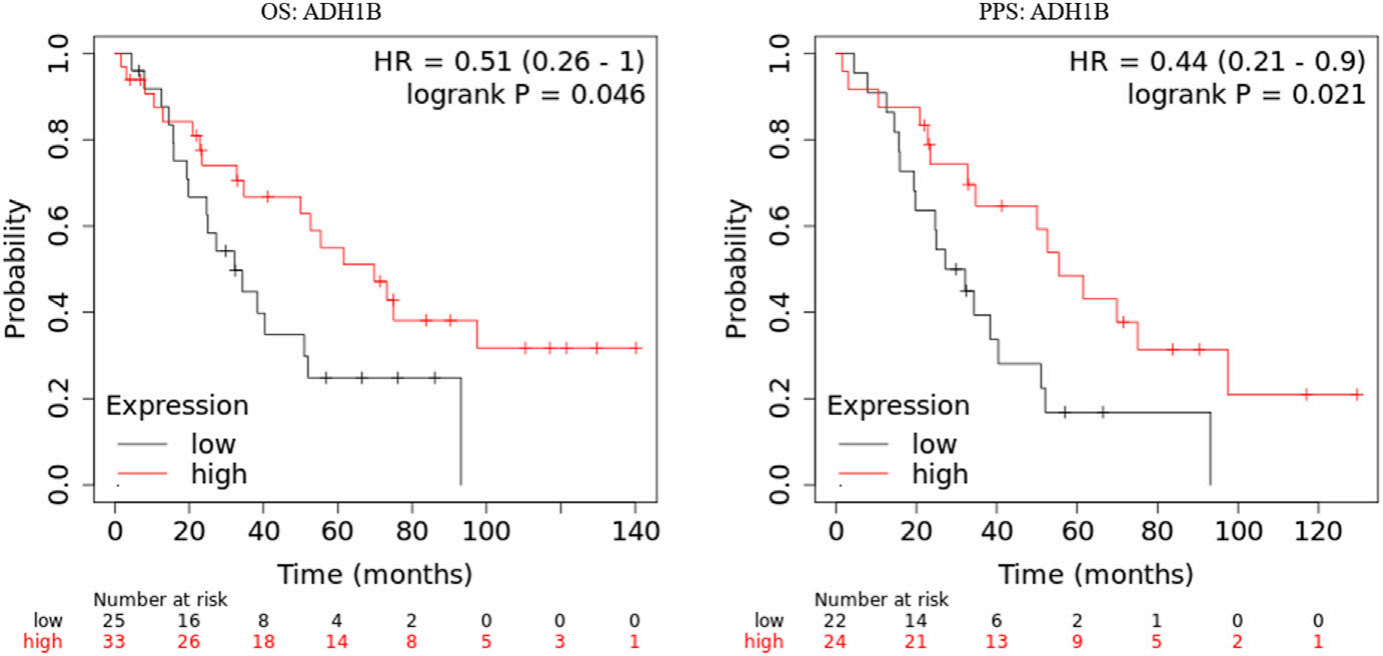
Kaplan-Meier curves of ADH1B in GSE30161. Kaplan-Meier plotter database was applied to obtain the OS and PPS curves of ADH1B in patients with ovarian cancer.

The prognostic values of ADH1B, AOX1, and DPYD were investigated in the GSE30161 dataset (Ferriss et al., 2012) through Kaplan-Meier curves. As shown in Figure 2, ovarian cancer patients with higher ADH1B expression have favorable OS and PPS. On the contrary, the expression of AOX1 and DPYD did not consistently affect the patients’ outcomes in the GSE30161 dataset (Supplementary Figures S1,S2). Therefore, these results brought ADH1B into sharper focus of further study.

### Investigations for Expression Level and Clinical Significance of ADH1B

**FIGURE 3:**
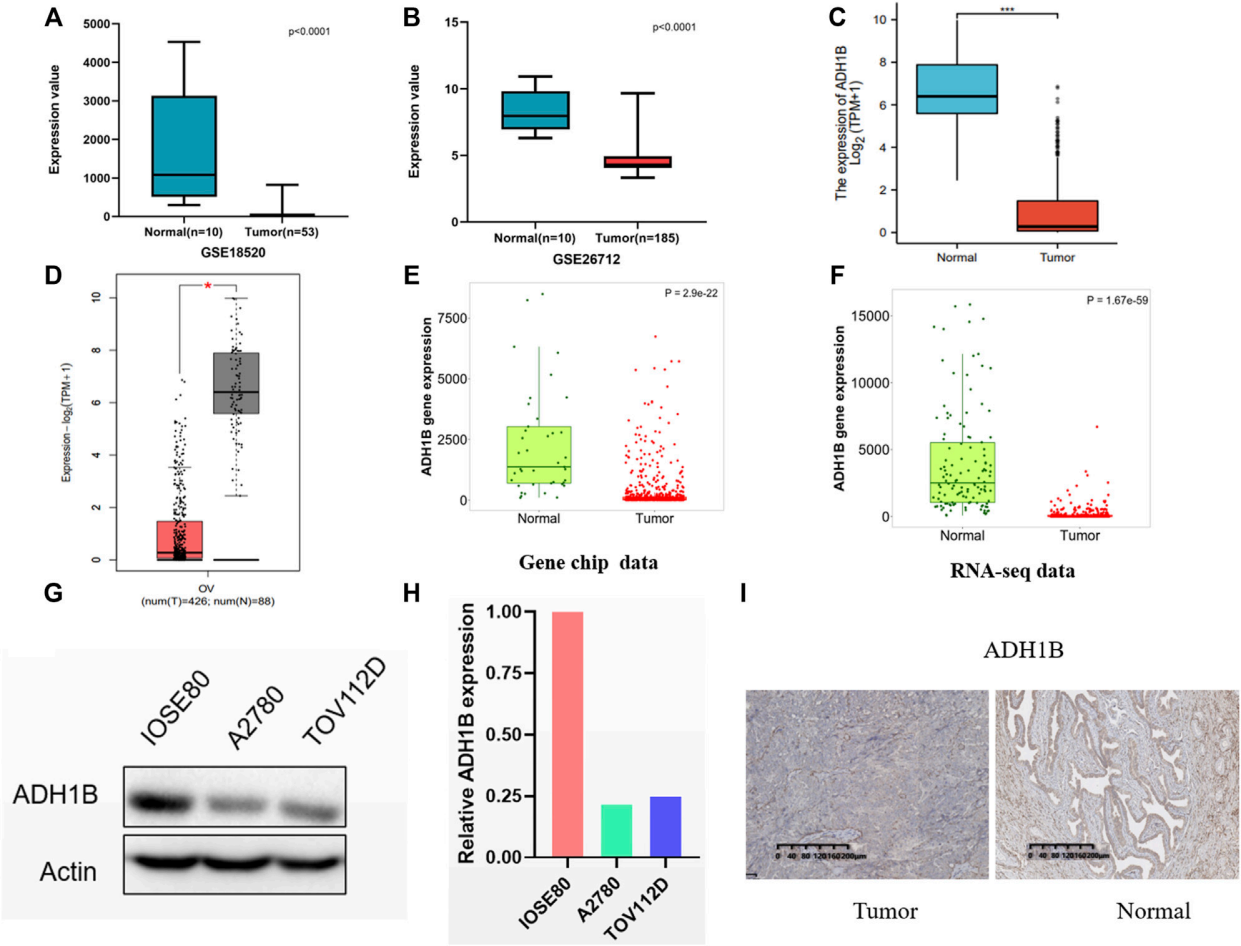
The expression of ADH1B in ovarian cancer tissues. **(A–D)** Compared with normal ovarian tissue, a significantly reduced ADH1B could be obtained in GSE18520 **(A)**, GSE26712 **(B)**, TCGA-OV **(C)**, and GEPTA2 **(D)**. **(E,F)** The verification of down-regulated expression level of ADH1B using TNMplot database from gene chip data **(E)** and RNA-seq data **(F)**. **(G,H)** ADH1B was downregulated in two OV cells, A2780 and TOV112D. **(I)** Immunohistochemical analysis indicated the downregulated ADH1B in OV patients. *p < 0.05; ***p < 0.001.

We compared the ADH1B expression between ovarian cancer tissue and normal tissues in GSE18520 and GSE26712, and found the reduced ADH1B expression in tumor tissues (Figures 3A,B). Then, data from TCGA-OV suggested that ADH1B expression is significantly down-regulated in ovarian cancer (Figure 3C). Furthermore, the lowly-expressed ADH1B in OV patients was also obtained from the GEPIA2 platform (*p* < 0.05) (Figure 3D). To enhance the credibility of these findings, the TNMplot was further applied to validate the decreased ADH1B in tumor groups from Gene-chip data (*p* = 2.9e-22) and RNA-seq data (*p* = 1.67e-59) (Figures 3E,F). Moreover, the downregulated ADH1B was further confirmed in two OV cells (A2780 and TOV112D) (Figures 3G,H) and OV tissues (Figure 3I).

To investigate the relationship between clinical characteristics and ADH1B expression, we divided ovarian cancer patients into two groups with high and low ADH1B expression and performed statistical analysis using the clinical data from the TCGA database. As shown in Table 1, in the “Fédération Internationale de Gynécologie et d’Obstétrique” (FIGO) stage, primary therapy outcome, race, histologic grade, and venous invasion, some differences were noticed but they did not achieve statistical significance. However, age, anatomic neoplasm subdivision, lymphatic invasion, tumor residual, and tumor status were all significantly different between the low ADH1B expression group and the high ADH1B expression group. These findings provided a novel and hopeful perspective on clinical biomarkers for ovarian cancer.

**TABLE 1:** The demographic parameters for ovarian cancer patients from the TCGA database based on the ADH1B levels.

Characteristic	Low expression of ADH1B	High expression of ADH1B	*P*-values
n	189	190	—
FIGO stage, n (%)	—	—	0.247
Stage I	1 (0.3%)	0 (0%)	—
Stage II	13 (3.5%)	10 (2.7%)	—
Stage III	151 (40.2%)	144 (38.3%)	—
Stage IV	23 (6.1%)	34 (9%)	—
Primary therapy outcome, n (%)	—	—	0.511
Progressive disease (PD)	12 (3.9%)	15 (4.9%)	—
Stable disease (SD)	9 (2.9%)	13 (4.2%)	—
Partial response (PR)	19 (6.2%)	24 (7.8%)	—
Complete response (CR)	114 (37%)	102 (33.1%)	—
Race, n (%)	—	—	0.289
Asian	7 (1.9%)	5 (1.4%)	—
Black or African American	16 (4.4%)	9 (2.5%)	—
White	160 (43.8%)	168 (46%)	—
Age, n (%)	—	—	0.015
< =60	116 (30.6%)	92 (24.3%)	—
>60	73 (19.3%)	98 (25.9%)	—
Histologic grade, n (%)	—	—	0.051
G1	0 (0%)	1 (0.3%)	—
G2	16 (4.3%)	29 (7.9%)	—
G3	167 (45.3%)	155 (42%)	—
G4	1 (0.3%)	0 (0%)	—
Anatomic neoplasm subdivision, n (%)	—	—	0.011
Unilateral	63 (17.6%)	39 (10.9%)	—
Bilateral	118 (33.1%)	137 (38.4%)	—
Venous invasion, n (%)	—	—	0.100
No	28 (26.7%)	13 (12.4%)	—
Yes	32 (30.5%)	32 (30.5%)	—
Lymphatic invasion, n (%)	—	—	0.023
No	34 (22.8%)	14 (9.4%)	—
Yes	50 (33.6%)	51 (34.2%)	—
Tumor residual, n (%)	—	—	0.015
No residual disease (NRD)	42 (12.5%)	25 (7.5%)	—
Residual disease (RD)	121 (36.1%)	147 (43.9%)	—
Tumor status, n (%)	—	—	0.034
Tumor-free	45 (13.4%)	27 (8%)	—
With tumor	126 (37.4%)	139 (41.2%)	—
Age, median (IQR)	57 (50, 67)	61 (52, 69.75)	0.041

### Co-Expression Analysis of ADH1B

**FIGURE 4:**
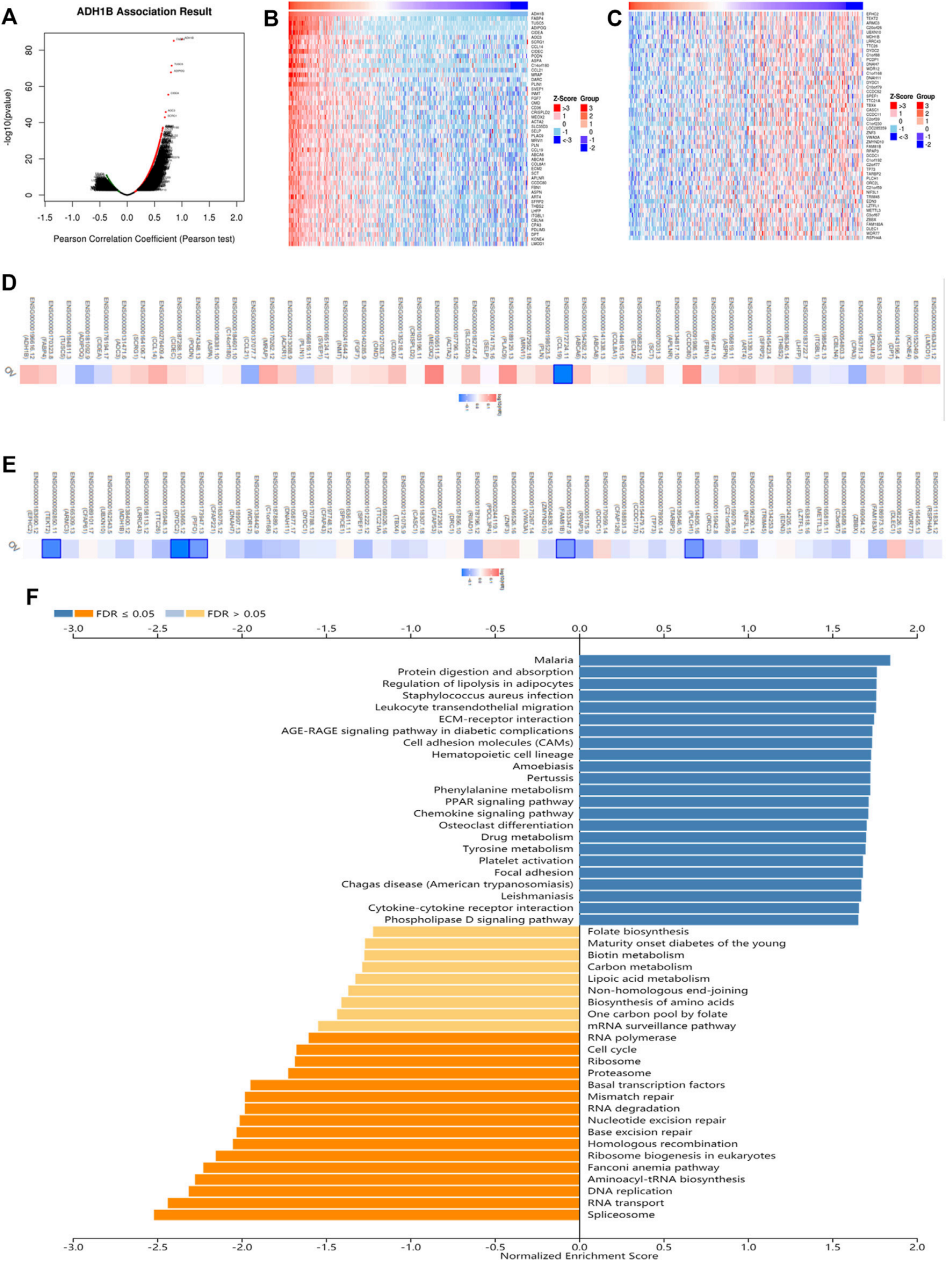
The co-expression analysis of ADH1B in ovarian cancer. **(A)** The identification of co-expressed genes remarkably related to ADH1B using the LinkedOmics database. **(B,C)** The top 50 genes positively and negatively in connection with ADH1B were shown on the heat map. Red indicated positive correlations while blue negative. **(D,E)** The top 50 genes having positive and negative associations with ADH1B were presented on the survival heat map. **(F)** Bar chart of the KEGG enrichment analysis of ADH1B.

In order to evaluate the biological functions of ADH1B in OV, the ADH1B co-expression pattern in the TCGA-OV cohort was considered an important event which was implemented by LinkedOmics. We observed that there were 3413 genes negatively associated with ADH1B and 3841 genes positively (Figure 4A). Then, the prognostic significance of partial genes having high relative coefficients was explored. The results were presented in the form of heatmaps (Figures 4B,C). It was worth noting that a protective hazard ratio was more likely to be owned by most negative co-expressed genes (34 out of 50) while an adverse hazard ratio by most positive co-expressed genes (32 out of 50) (Figures 4D,E). Meanwhile, the KEGG Pathway and GO enrichment analysis of ADH1B were conducted. As shown in Figure 4F, spliceosome, RNA transport, DNA replication, and aminoacyl-tRNA biosynthesis were the top four enriched KEGG pathways. Ribonucleoprotein complex localization, spliceosomal complex, and helicase activity were the top enriched terms in biological process, cellular component, and molecular function (Supplementary Figures S3A–C).

### Roles of ADH1B in Immune Regulation

**FIGURE 5:**
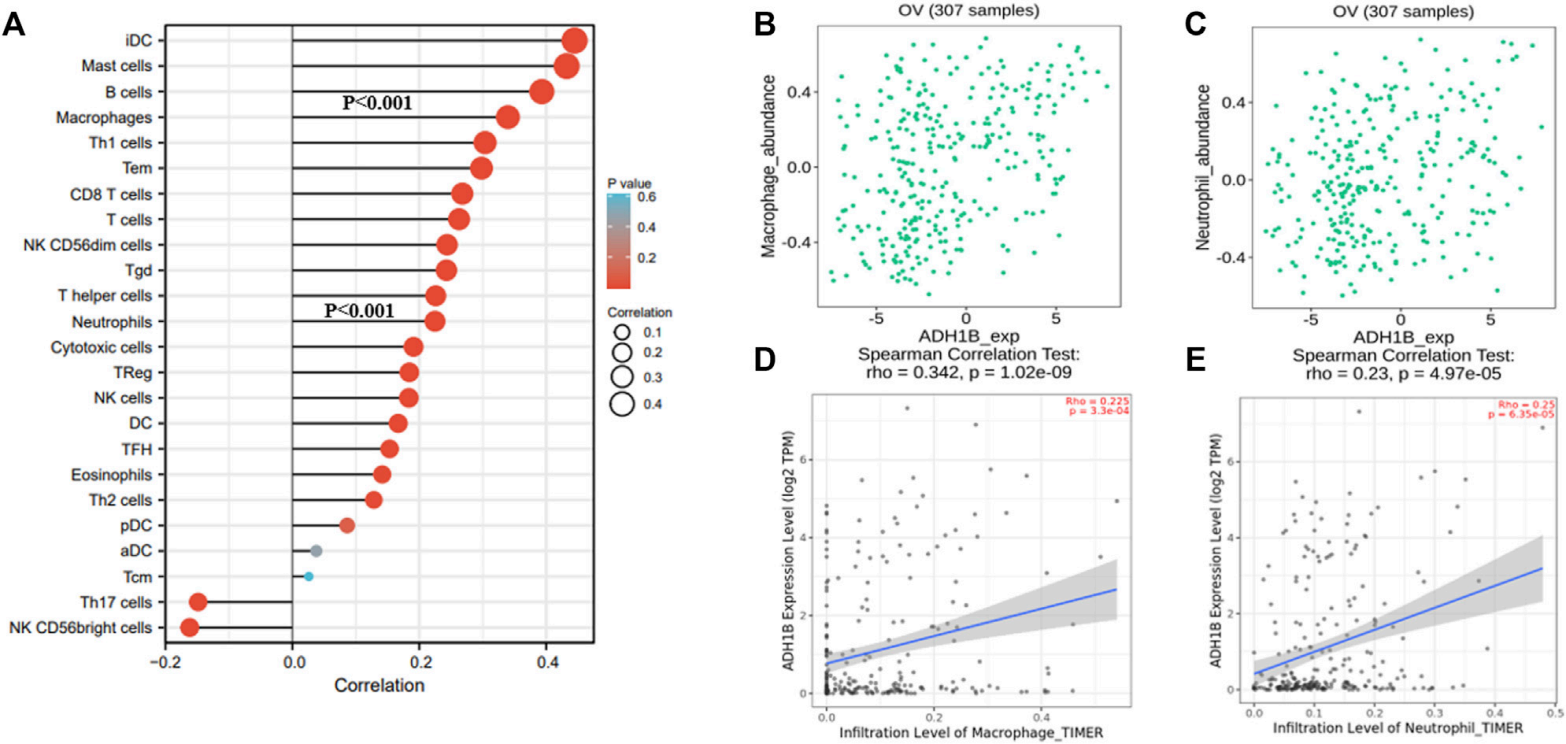
The relationship between ADH1B and immune infiltration. **(A)** 24 kinds of immune-infiltrating cells related to ADH1B expression. **(B–E)** Scatter plots were drawn through TIMER2.0 and TISIDB database to validate the correlations between ADH1B and immune-infiltrating cells, including macrophages and neutrophils.

A survey was carried out for the relationship between ADH1B and immune infiltration cells following these steps. We primarily evaluated the immune infiltration cells related to ADH1B in ovarian cancer through the ssGSEA algorithm with Pearson correlation. The results revealed that iDC, mast cells, B cells, macrophages, Th1, Tem, CD8 T-cells, T-cells, NK CD56dim cells, Tgd, T-helper cells, neutrophils, and cytotoxic cells were a positive correlation with ADH1B expression level (*p* < 0.001) (Figure 5A). Subsequently, TISIDB and TIMER databases were used for further validation. We finally concluded that macrophages and neutrophils were positively correlated with the expression of ADH1B (*p* <0.001) (Figures 5B–E).

In order to improve our understanding of the role of ADH1B in immune regulation, we shifted the focus of our research to the relationships between ADH1B between immunostimulators, immunoinhibitors, chemokines, and receptors. As shown in Supplementary Figures S4A–B, CD96 (Spearman r = 0.329, *p* = 4.26e-09), CD244 (Spearman r = 0.318, *p* = 1.5e-08), KDR (Spearman r = 0.352, *p* = 2.74e-10), and TGFB1 (Spearman r = 0.312, *p* = 2.66e-08) were the top four prominent markers of immune stimulation. Conversely, CXCL12 (Spearman r = 0.486, *p* = 2.22e-16), NT5E (Spearman r = 0.415, *p* = 2.19e-14), TNFRSF17 (Spearman r = 0.383, *p* = 5.4e-12), and TNFSF4 (Spearman r = 0.355, *p* = 1.96e-10) were the top four prominent markers of immune suppression. These findings indicated that ADH1B expression had a significantly positive relation with the molecules mentioned earlier. Supplementary Figure S5A suggested that the association between ADH1B expression and chemokines in patients with ovarian cancer, the result reported that the four most significant chemotactic agents were CCL21 (Spearman r = 0.629, *p* < 2.22e-16), CCL14 (Spearman r = 0.58, *p* < 2.22e-16), CCL19 (Spearman r = 0.518, *p* < 2.22e-16), and CCL21 (Spearman r = 0.486, *p* < 2.22e-16). Supplementary Figure S5B showed the most remarkable receptors closely related to ADH1B expression, CCR2 (Spearman r = 0.383, *p* = 5.4e-12), CCR4 (Spearman r = 0.355, *p* = 1.96e-10), CCR5 (Spearman r = 0.325, *p* = 6.77e-09), and CCR7 (Spearman r = 0.339, *p* = 1.4e-09) were included.

Overall, the implications of these results were that ADH1B was demonstrated to participate in a variety of immune regulation in ovarian cancer and able to be a new breakthrough in the treatment of ovarian cancer.

### The Potential Therapeutic Implications of ADH1B in Ovarian Cancer

**FIGURE 6:**
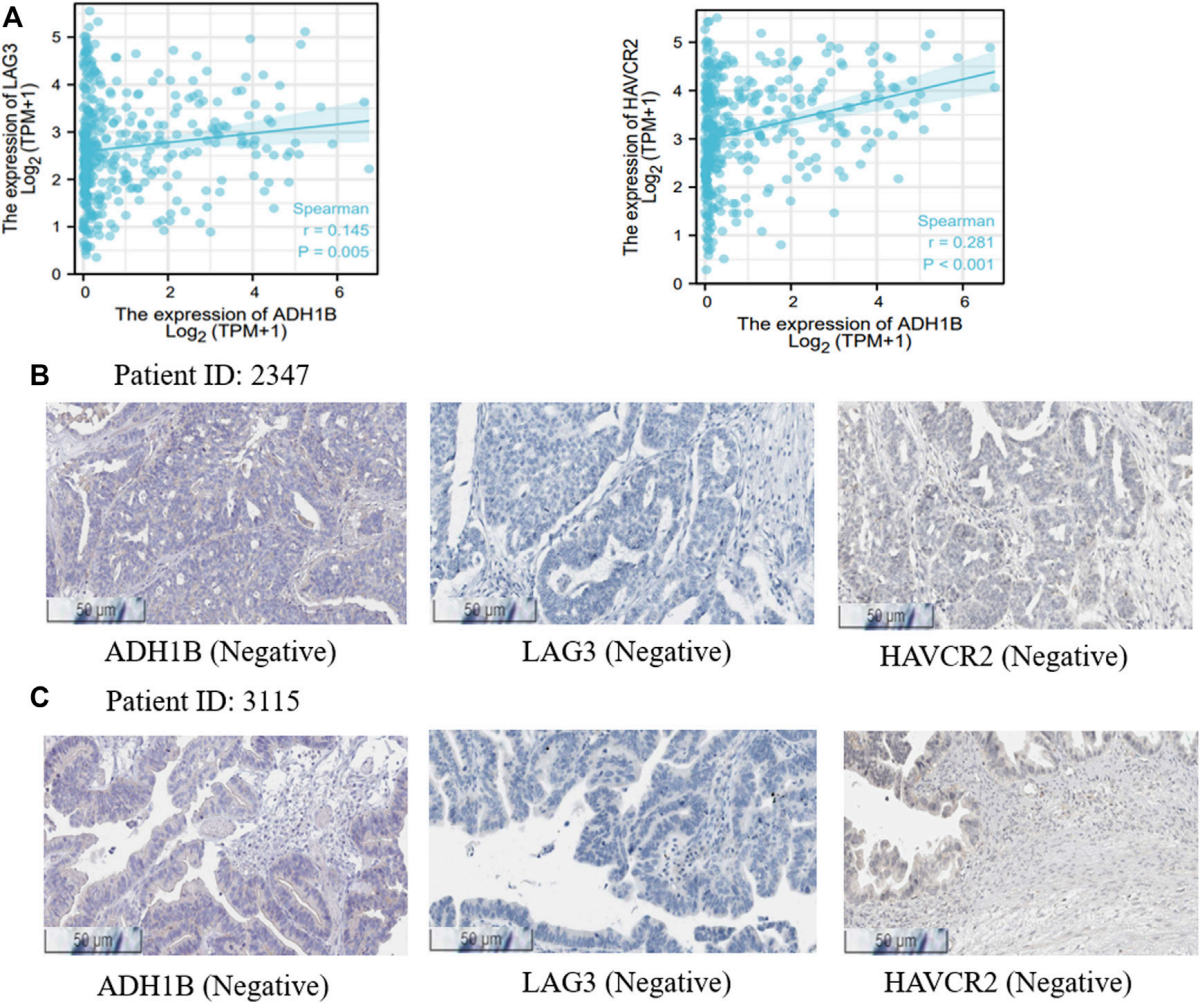
Treatment correlation analysis of ADH1B. **(A)** The associations between ADH1B with LAG3 and HAVCR2. **(B,C)** the Human Protein Atlas portal validated the downregulated ADH1B, LAG3, and HAVCR2 in OV tissues.

It is well known that chemotherapy is an important strategy for OV patients. Cyclophosphamide is one of the most used chemotherapeutic drugs (Ray-Coquard et al., 2020; Zsiros et al., 2021). Expectedly, we found that the expression of ADH1B was negatively associated with the activity of cyclophosphamide (Pearson r = -0.88, *p* = 0.047) (Supplementary Figure S6), indicating that high expression of ADH1B had an adverse effect on the chemotherapeutic effect. Additionally, positive correlations could be observed between ADH1B expression and multiple targets for immunotherapy, such as LAG3 and HAVCR2 (Figure 6A). Then, we employed the Human Protein Atlas portal (https://www.proteinatlas.org/) to validate the positive association between ADH1B and LAG3/HAVCR2. As shown in Figures 6B,C, among the 11 cases of OV tissues examined for ADH1B staining, six cases had negative expression and three cases had weak intensity. Meanwhile, all of the 11 cases displayed negative intensity for LAG3 and HAVCR2 staining. All these findings together suggested that ADH1B, LAG3, and HAVCR2 were significantly down-regulated in cancer tissues, which would offer a good prospect that targeted ADH1B could increase the therapeutic efficacy of OV patients.

## Discussion

In this research, our aim is to find a candidate prognostic and targeted biomarker for the treatment of ovarian tumors. As a result, three down-regulated genes, ADH1B, AOX1, and DPYD, were identified using multiple bioinformation databases and drug metabolism-related genes datasets. Then, a series of survival analyses were conducted and ADH1B was the most significant biomarker. Moreover, we analyzed the co-expressed genes of ADH1B and found that these genes positively or negatively correlated to ADH1B might have a considerable influence on prognosis and treatment of ovarian cancer. In addition, ADH1B has shown great importance in the immune microenvironment in ovarian cancer. Hence, we conducted that ADH1B was allowed to function as a biomarker aiding in the treatment and prognosis of ovarian cancer.

Most human diseases, including cancer, have something to do with multiple genes and genetic variations. Similarly, the metabolic effects of most drugs are involved in polygenic products (Relling and Evans, 2015). There are several studies indicating the significance of drug metabolism-related genes and the outcome of different cancers. For instance, He et al. (2020) reported that there was a worse prognosis in both poor and ultrarapid CYP2D6 metabolizers of tamoxifen in comparison with normal metabolizers in breast cancer (He et al., 2020). Moore et al. (2018) proved that BRCA1/2 mutation carriers reap greater benefits from olaparib maintenance therapy in ovarian cancer (Moore et al., 2018). Additionally, UGT1A1 polymorphism influenced clinical outcome in patients undergoing cytarabine chemotherapy for moderate critical myeloid leukemia (Díaz-Santa et al., 2020). The evidence presented that drug metabolism-related genes are crucial for personalized cancer therapy to get a better outcome for patients. However, there have been few reports of the relationship between drug metabolism-related genes and prognosis in ovarian cancer patients. Based on acquired chemotherapy resistance in ovarian cancer (Christie and Bowtell, 2017), it is necessary to explore new therapeutic approaches from a pharmacogenomics perspective and find novel molecular biomarkers or drug targets.

Aberrantly expressed ADH1B could participate in the regulation of cellular metabolism and drug metabolism (Li et al., 2018). ADH1B can be considered as a molecular biomarker of residual disease and its overexpression promotes invasion and metastasis in ovarian cancer (Tucker et al., 2014). Moreover, the activity of the ADH1B is significantly increased in the OV group compared with normal ovarian tissues. As a result, increased acetaldehyde concentration induced by activated ADH1B produced carcinogenic effects in ovarian cells (Orywal et al., 2013). These findings may explain the roles of ADH1B in the development and progression of ovarian cancer. In addition, the higher ADH activity was observed in ESCC and bladder cancer. Zhang et al. (2010) found that the Han Chinese with ADH1B His47Arg locus have greater interaction with alcohol consumption and are prone to develop ESCC (Zhang et al., 2010). Masaoka et al. (2016) proved that the risk of bladder cancer was greatly increased in people with ADH1B Arg + due to gene-environment interaction (Masaoka et al., 2016). Therefore, ADH1B has a significant impact on tumorigenesis and progression. In our study, ADH1B, a drug metabolism-related gene, was down-regulated in ovarian cancer tissues, which indicated a better life span expectancy.

The results of KEGG annotation were shown in Figure 4F. Spliceosome, RNA transport, and DNA replication were the top three signal pathways regulated by ADH1B co-expressed genes in OV patients. The spliceosome is a powerful tool for removing introns from pre-mRNA molecules (Galej et al., 2014). Several splicing factors, including catenin *β*-like 1(CTNNBL1) and ubiquitin-specific peptidase 39 (USP39), could promote the growth and invasion of OV cells (Li et al., 2017; Wang et al., 2021). The intranuclear genetic information could be transferred into the cytoplasm through RNA transport, participating in cancer pathogenesis and therapeutic response (Gasparski et al., 2022). DNA replication is a key step in maintaining genome stability and is frequently disrupted in cancer cells. Inhibitors of poly (ADP-ribose) polymerase (PARP) targeting DNA repair were effective in treating patients with advanced OV (Yan et al., 2020).

Tumor microenvironment (TME) is a hot topic in recent years. Evidence has increasingly shown that tumor development, recurrence, and metastasis is in close relationship with the immune microenvironment as an acritical part of the tumor microenvironment (Fu et al., 2021). Meanwhile, a large number of studies on the immune microenvironment have contributed to the development of immunotherapy strategies and have increased the likelihood of favorable patient outcomes (Li et al., 2021). Ovarian cancer is usually found to be advanced and is prone to recurrence after surgery or chemotherapy, which leads to a high death rate (Lheureux et al., 2019) and survival for ovarian cancer has not improved significantly for decades (Torre et al., 2018). To improve the prognosis of ovarian cancer patients, special immunotherapy and developing new biomarkers become the development direction of therapeutic strategies now and even for a long time to come. At present, the major immunotherapies include oncolytic virus therapies, cancer vaccines, cytokine therapies, adoptive cell transfer, and immune checkpoint inhibitors which maybe be the new technologies and prospects for the treatment of ovarian cancer (Zhang and Zhang, 2020). Actually, the United States Food and Drug Administration recently has approved several immune checkpoint inhibitors for a variety of cancers, unfortunately not ovarian cancer (Odunsi, 2017). In our work, a comprehensive evaluation between ADH1B and the immune environment was conducted. The results suggested that the expression level of ADH1B had a positive relationship with macrophages and neutrophils. There has been a great deal of research indicating a connection between ovarian cancer and tumor-infiltrating immune cells including macrophages and neutrophils (Lee et al., 2019; Etzerodt et al., 2020). Macrophages can promote the invasion and chemoresistance of ovarian cancer (Raghavan et al., 2019). Neutrophils can release oxidized lipids to reactivate dormant tumor cells, resulting in early cancer recurrence (Perego et al., 2020). With a deeper understanding of the immune environment, the researchers have come up with various therapeutic ideas. Barkal et al. (2019) found that blocking the CD24 signaling pathway mediated by macrophage siglec-10 is promising as a new target of immunotherapy (Barkal et al., 2019). Raghavan et al. (2019) thought that the WNT pathway which promotes the interaction between ovarian cancer stem cells (CSC) and macrophages is a potential target for reducing the aggressiveness of ovarian cancer (Raghavan et al., 2019). Additionally, the upregulation of PD-L1 expressed in neutrophils leads to accelerate immune escape of ovarian cancer cells which can be solved by targeting HOXA transcript at the distal tip (HOTTIP) (Shang et al., 2019). Based on our research, there is reason to believe that ADH1B may be a potential treatment strategy with the regulation of macrophages and neutrophils for ovarian cancer.

Furthermore, the most valuable immunostimulators (CD96, CD244, KDR, and TGFB1), immunoinhibitors (CXCL12, NT5E, TNFSRF17, and TNFSF4), chemokines (CCL21, CCL14, CCL19, and CCL12), and chemokine receptors (CCR2, CCR4, CCR5, and CCR7) related to ADH1B were obtained. There has been a lot of research on these molecules and ovarian cancer. CD96 has been proved the key for natural killer (NK) cell reactivity to advanced ovarian cancer (Maas et al., 2020). TGFBI produced by macrophages can conduce to suppress the immune microenvironment of ovarian cancer (Lecker et al., 2021). The CXCL12 has been seen as a strong accelerant to promote metastasize and invasion in ovarian cancer (Luo et al., 2019). CCL21 has been shown an association with bad OS in TP53 wild-type serous ovarian cancer (Ignacio et al., 2018). CCR2 might make a difference in enhancing the ability of ovarian cancer cell peritoneal metastasis and portending a poor prognosis (Sun et al., 2020). Generally, these studies show that ADH1B is a key part of the immune microenvironment and regulates a variety of immune cells against ovarian cancer cells. On this basis, ADH1B may be a potential therapeutic target and contribute to the treatment of ovarian cancer.

## Conclusion

In summary, the downregulation of ADH1B was illustrated in this study, which was shown with a good prognosis value for ovarian cancer. In addition, the expression of ADH1B has a significant correlation with the immune microenvironment. Immunostimulators, immunoinhibitors, chemokines, and chemokine receptors were included. Consequently, ADH1B could be significant for immune infiltration and be valuable in guidelines for treatment and evaluation of prognosis in patients with ovarian cancer. It was concluded that ADH1B might provide a new perspective for the treatment of ovarian cancer.

## Data Availability

The datasets presented in this study can be found in online repositories. The names of the repository/repositories and accession number(s) can be found in the article/Supplementary Material.
