# Supplementary figures and images for: The Roles of Drug Metabolism-Related ADH1B in Immune Regulation and Therapeutic Response of Ovarian Cancer
Source: Front Cell Dev Biol. 2022 Jun 9;10:877254. doi: 10.3389/fcell.2022.877254 (PMC9218672; doi:10.3389/fcell.2022.877254)

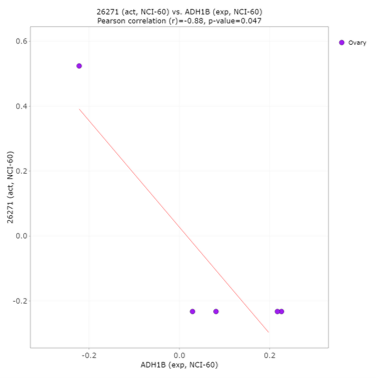

Supplement: Supplementary file 1 [file Image6.TIF]

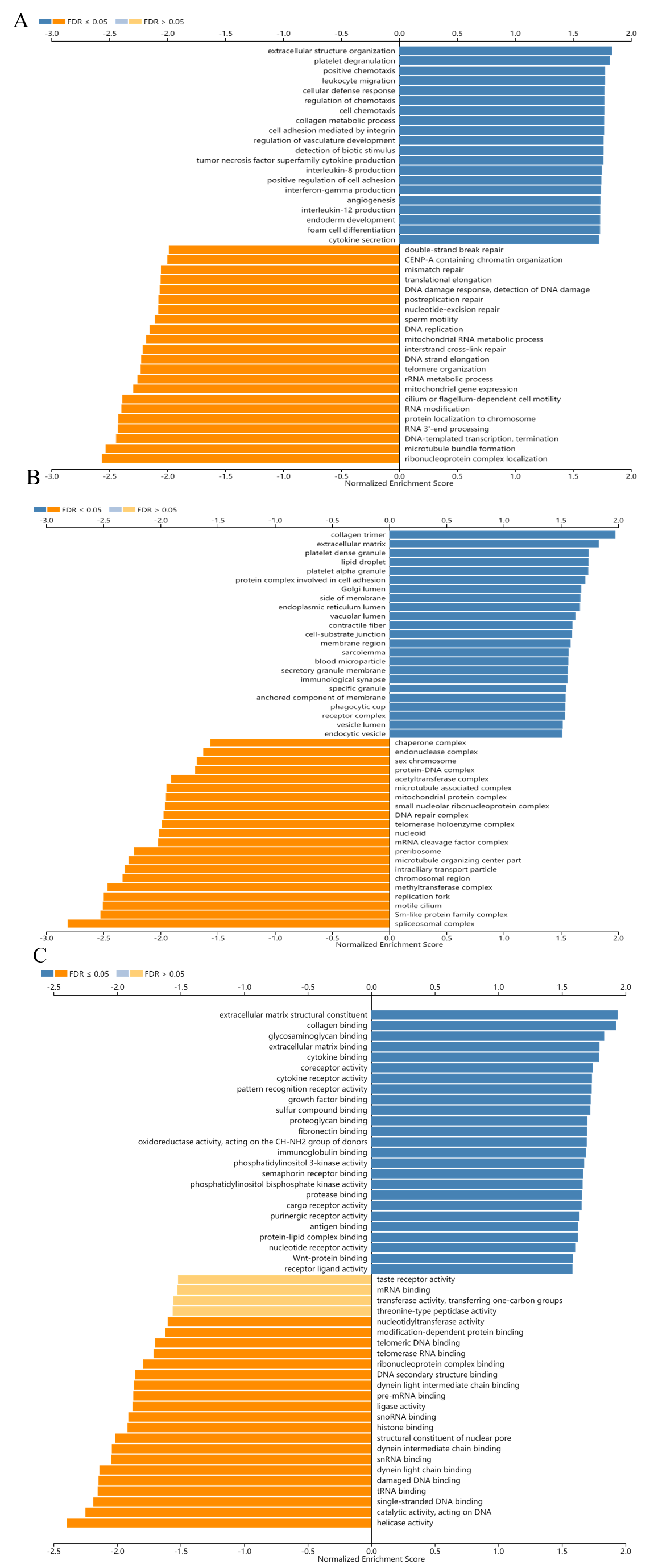

Supplement: Supplementary file 2 [file Image3.TIF]

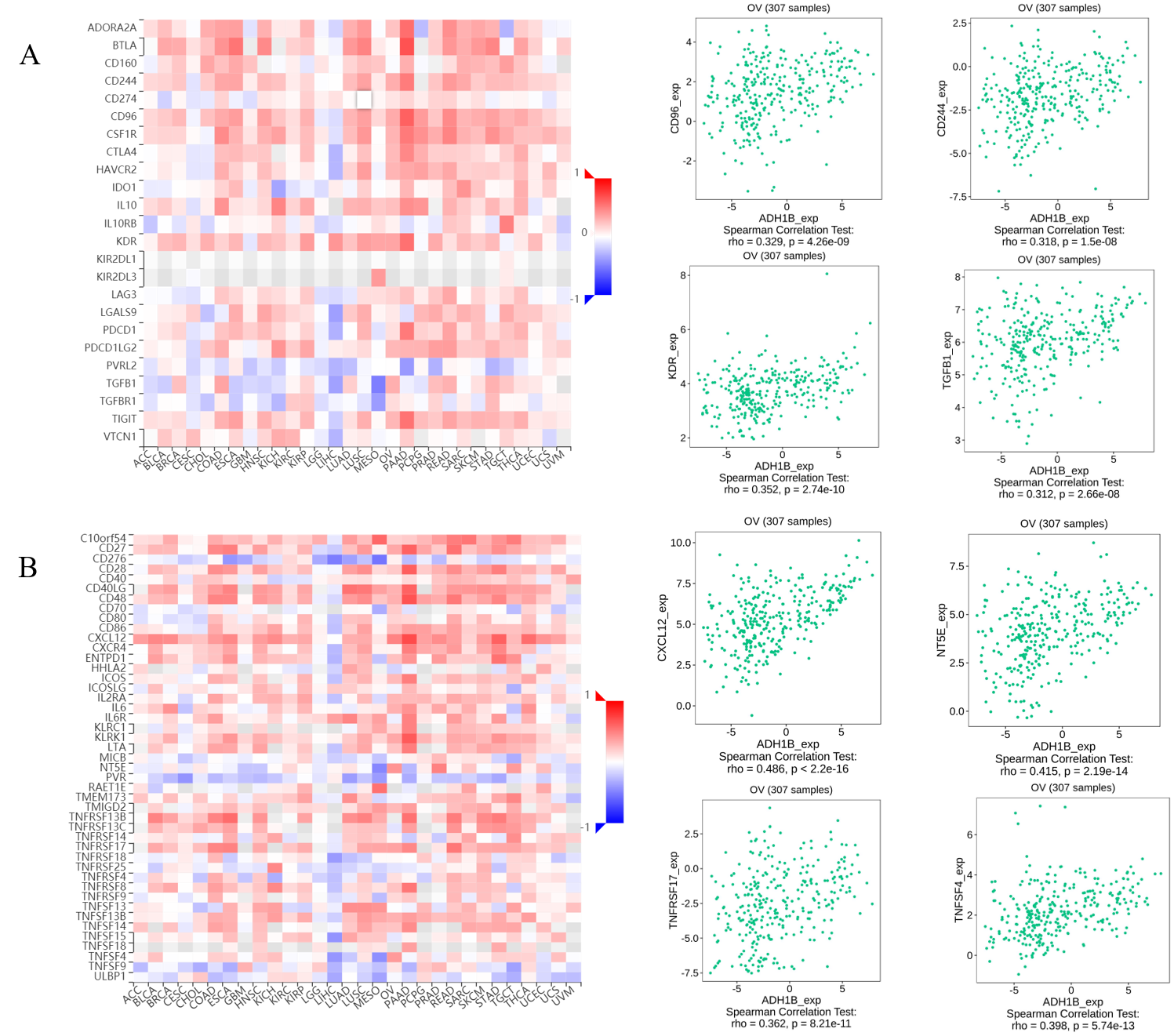

Supplement: Supplementary file 3 [file Image4.TIF]

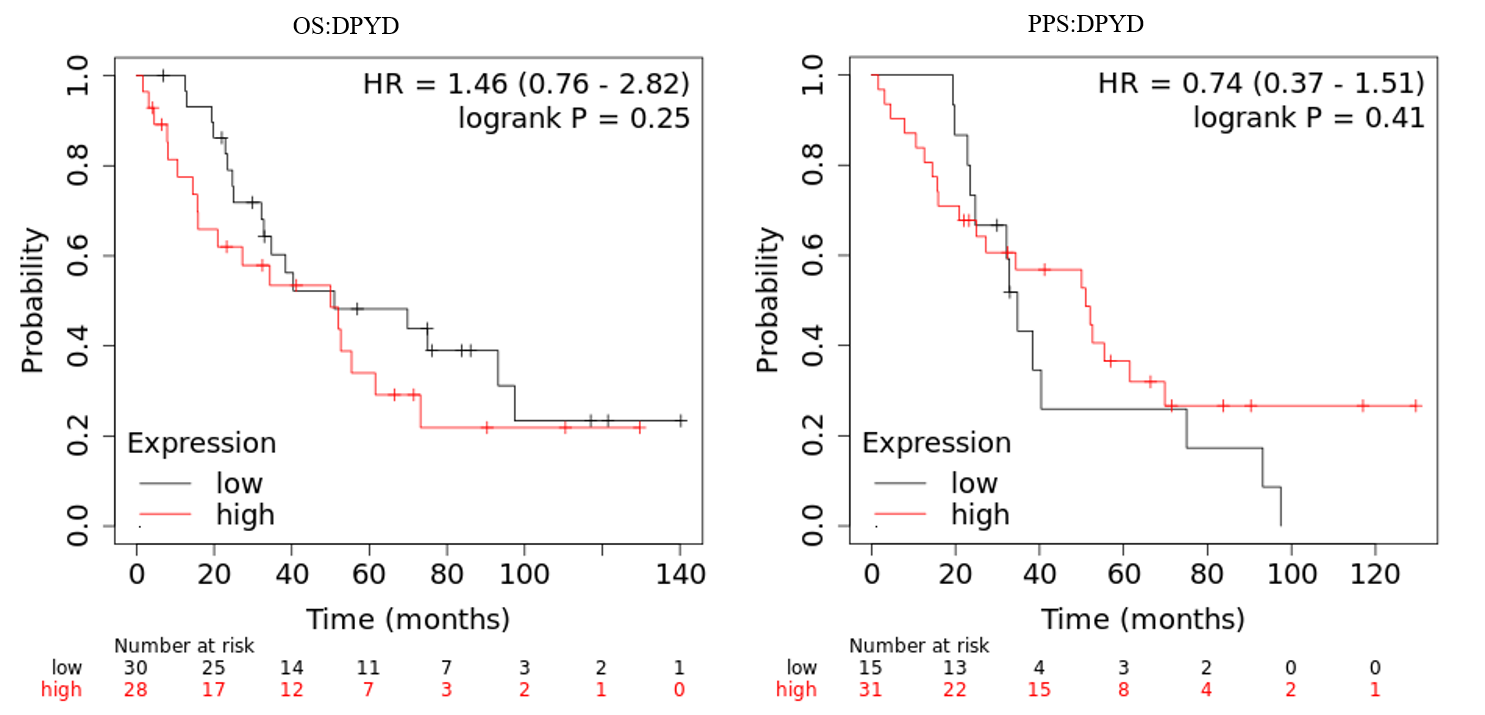

Supplement: Supplementary file 4 [file Image2.TIF]

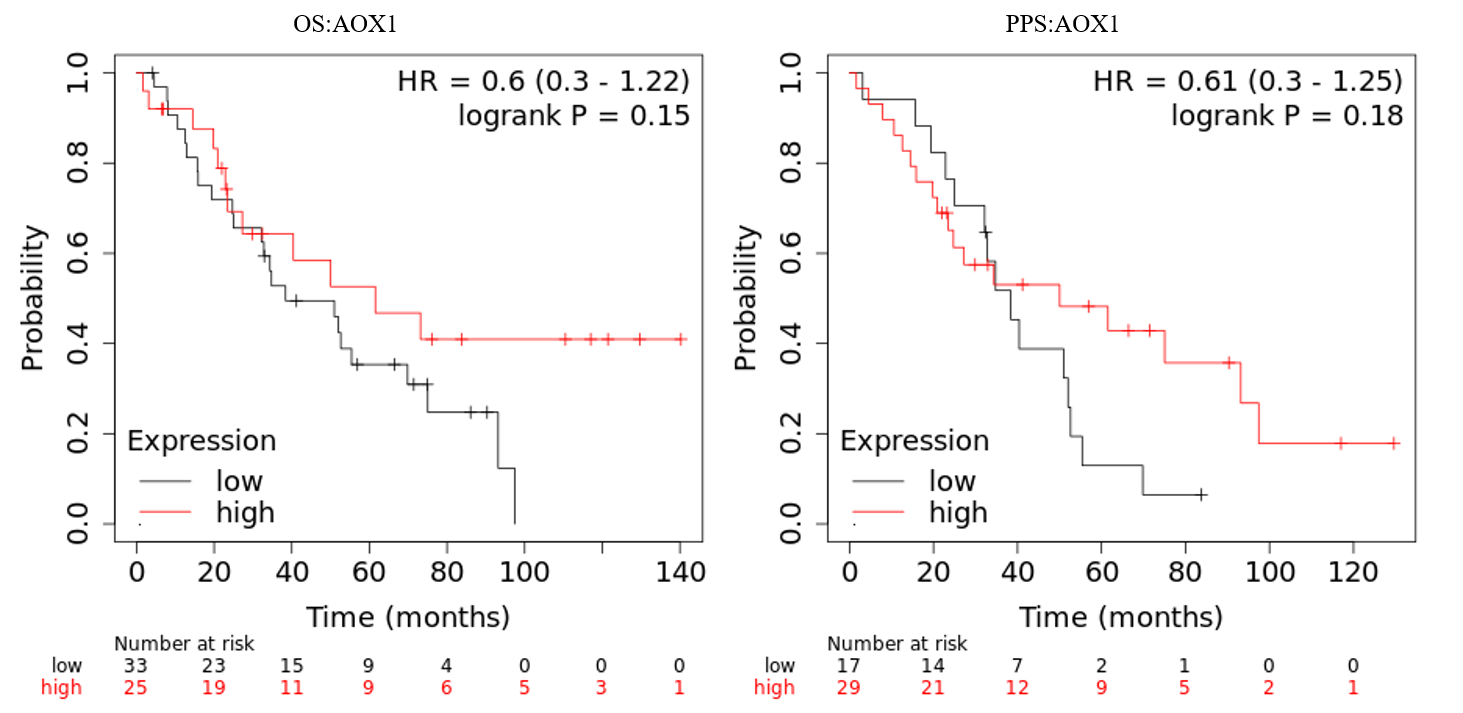

Supplement: Supplementary file 5 [file Image1.TIF]

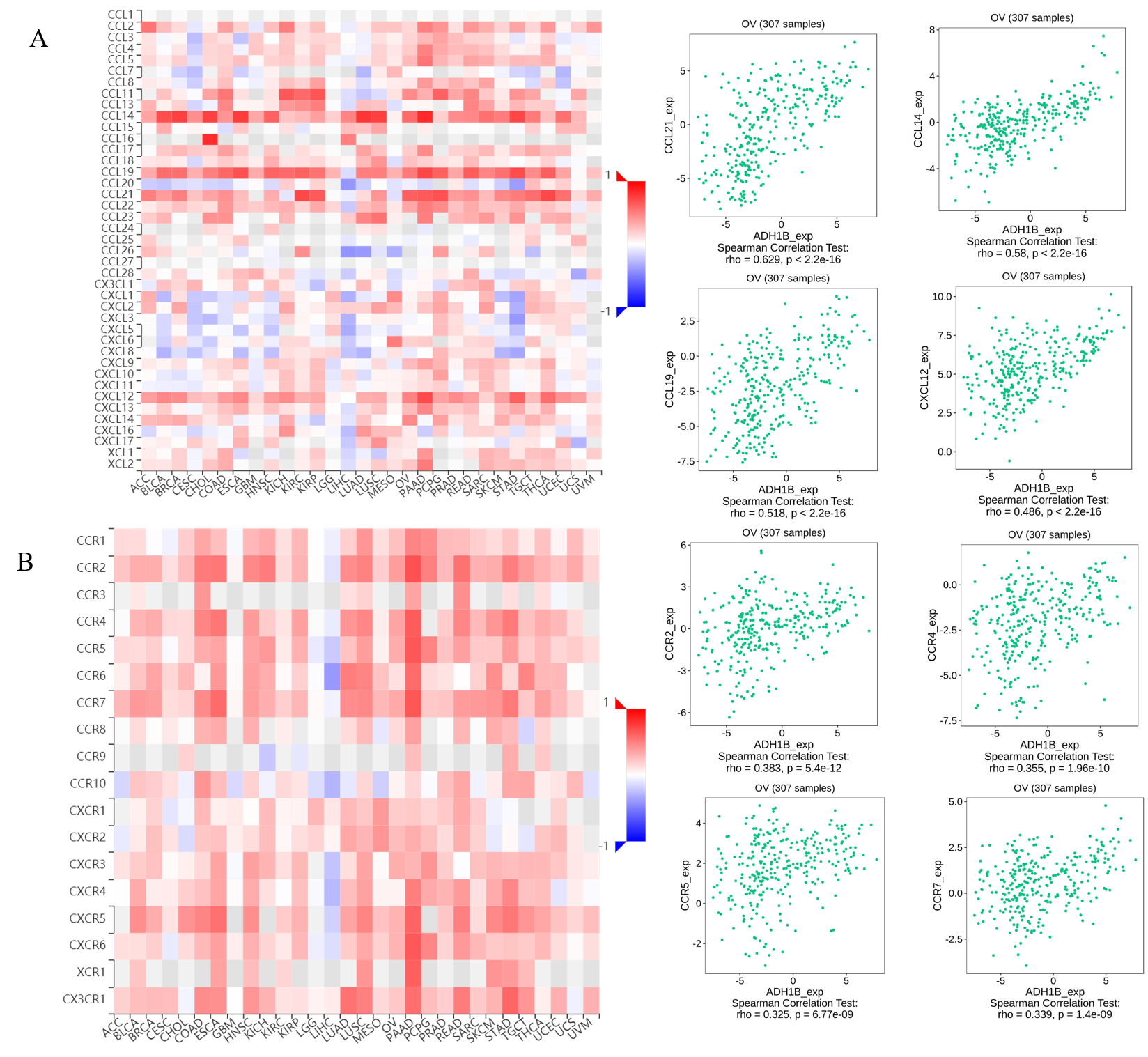

Supplement: Supplementary file 6 [file Image5.TIF]
